# 20 Assessing Mental Health’s Impact on Pain and Itch Perception in Post-Burn Hypertrophic Scars

**DOI:** 10.1093/jbcr/iraf019.020

**Published:** 2025-04-01

**Authors:** Casey Meretta, Lia Mandavalli, Ishaan Nandwani, Taryn Travis, Shawn Tejiram, Jeffrey Shupp, Daniel Schneider, Bonnie C Carney

**Affiliations:** The Burn Center at MedStar Washington Hospital Center; Nova Southeastern University; Georgetown University School of Medicine; MedStar Washington Hospital Center; MedStar Washington Hospital Center; MedStar Washington Hospital Center; MedStar Washington Hospital Center; MedStar Washington Hospital Center

## Abstract

**Introduction:**

Burn injuries often result in hypertrophic scars (HTS) associated with pain and itch (P&I). Laser scar revision (FLSR) has become a popular adjunct to existing treatments for HTS, yet some patients continue to experience P&I, severely impairing quality of life.

Mental health disorders can greatly impact patient perception of P&I. This study assessed the influence of mental health on P&I perceptions in post-burn FLSR patients, hypothesizing that patients with no mental health diagnosis would improve more rapidly than those with a diagnosis during laser therapy.

**Methods:**

Demographics (age, sex, Fitzpatrick skin type, race) and scar characteristics (scar age, burn etiology, location, size) were obtained. Patients (n=110) were split into two cohorts, those with no mental health diagnosis, (NoMH, n=53) and those with (MHD, n=57). Inclusion in MHD required a preexisting diagnosis or one from inpatient burn psychology. All patients received a preoperative evaluation (pre-FLSR), at least 3 treatments (FLSR 1, 2, 3) and evaluation via patient and observer scar assessment scale (POSAS) -observer(-O), and -patient(-P). All P&I scores were obtained from the POSAS-P evaluations.

**Results:**

Intergroup comparisons between P&I scores and FLSR sessions were not significant. However, intragroup analysis of MHD revealed significant decreases in pain between pre-FLSR and post-FLSR session 1 (p< 0.001), FLSR 2 (p< 0.05) and FLSR 3 (p< 0.01). In NoMH the only decrease was from pre-FLSR and FLSR 3 (p< 0.01). Itch scores decreased in MHD post-FLSR 2 (p <.05) and FLSR 3 (p <.001) compared to pre-FLSR. NoMH had no differences between pre- and post-laser itch. There were no differences between group POSAS-O average scores. For NoMH, there were decreased scores at post-FLSR 2 (p< 0.01) and FLSR 3 (p< 0.001) and in MHD at post-FLSR 2 (p< 0.001) and FLSR 3 (p< 0.0001). There were no significant differences in concomitant medications between the MHD and NoMH groups except related to SSRIs.

**Conclusions:**

Contrary to the hypothesis, MHD patients reported a more significant decrease in P&I throughout FLSR treatment compared to NoMH. This is despite POSAS-O scores demonstrating no differences between groups in observer-reported scar healing and suggests a factor outside of scar healing impacts P&I perception.

**Applicability of Research to Practice:**

Interventions implemented by the burn psychology team are a likely cause of the improved scar perception in MHD patients. The data further shows the importance of a dedicated burn psychology team and warrants further studies on the influence of psychological support on post-burn patient outcomes in the setting of laser.

**Funding for the Study:**

This work was funded in part by the National Center for Advancing Translational Science (NCATS/NIH)

